# Subdural Hygroma: A Morbid Complication of Intrathecal Chemotherapy in a Patient with B-Cell Acute Lymphocytic Leukemia

**DOI:** 10.1155/2019/3494056

**Published:** 2019-09-30

**Authors:** Mina Shenouda, Madhulika Urella, Jennifer Dotson, Hassaan Yasin, Yehuda Lebowicz

**Affiliations:** ^1^Marshall University, Department of Hematology and Oncology, 1400 Hal Greer Blvd, Huntington, WV 25701, USA; ^2^Marshall University, Department of Internal Medicine, 1249 15th St., Suite 2000, Huntington, WV 25701, USA

## Abstract

A subdural hygroma is an accumulation of cerebrospinal fluid in the subdural space that may occur secondary to trauma and surgery, or for iatrogenic reasons, such as a lumbar puncture. Lumbar puncture is a procedure used commonly for intrathecal chemotherapy for patients with B-cell acute lymphocytic leukemia (B-ALL) though subdural hygroma is a very rare complication. We present a case of a fatal, refractory subdural hygroma in a patient with B-ALL.

## 1. Introduction

Subdural hygroma is an uncommon complication after lumbar puncture. A subdural hygroma is the accumulation of cerebrospinal fluid (CSF) in the subdural space that may occur for a number of reasons. Subdural hygromas are encountered in all age groups. Subdural hygroma affects the critical age groups of less than 5 years and more than 60 years, when the dural space is large enough for the fluid to accumulate [1]. The demographics vary based on the underlying etiology: it may occur from trauma and iatrogenic causes related to spontaneous intracranial hypotension or idiopathic. Patients vary in clinical presentation, ranging from asymptomatic to symptomatic with headache, changes in mental status, nausea and vomiting, focal neurological deficits, and seizures [2]. The pathogenesis of subdural hygromas is not entirely understood. The most commonly encountered explanation is a tear in the arachnoid layer forming a ball valve opening allowing CSF a one-way passage into the subdural space. It has been proposed that subdural hygromas represent prominent subdural effusions in which there is a separation of the dural border cell layer with an accumulation of the fluid [1, 3]. Intrathecal chemotherapy (IT) is administered by performing a lumbar puncture and is widely used in hematological and systemic malignancies for either prophylactic or therapeutic intentions. We present a case of bilateral subdural hygroma as a rare complication of intrathecal methotrexate administration.

## 2. Case Presentation

**Figure 1:**
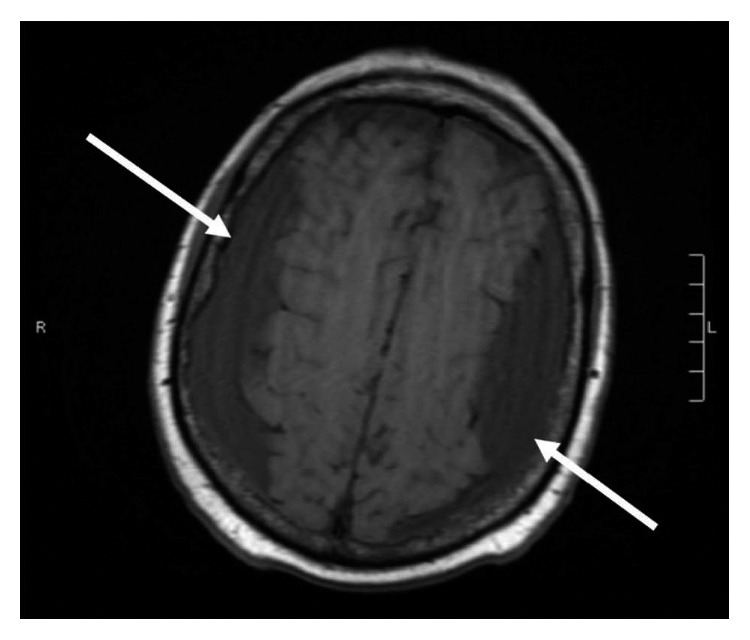
Axial T1 sequence from magnetic resonance imaging (MRI) of the brain.

**Figure 2:**
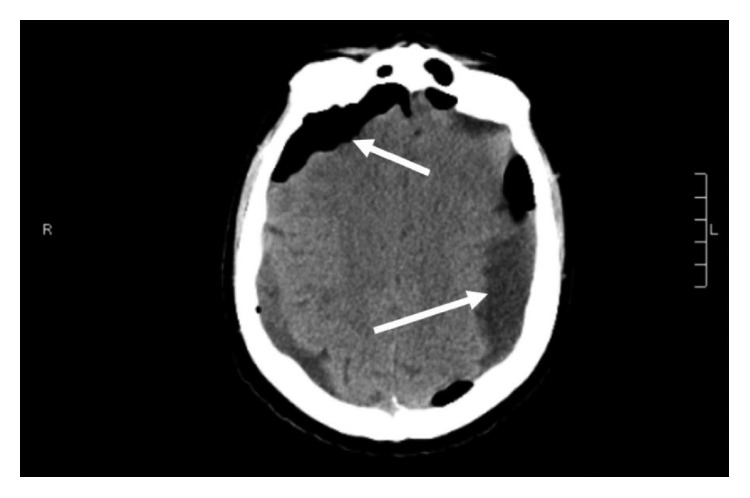
Computed tomography (CT) imaging of the brain after the bilateral burr hole procedure.

**Figure 3:**
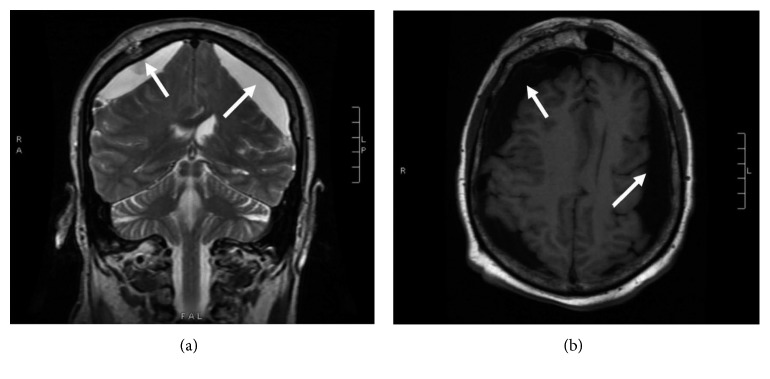
(a) T2-weighted coronal sequence from an MRI of the brain. (b) An axial T1 flair image.

**Figure 4:**
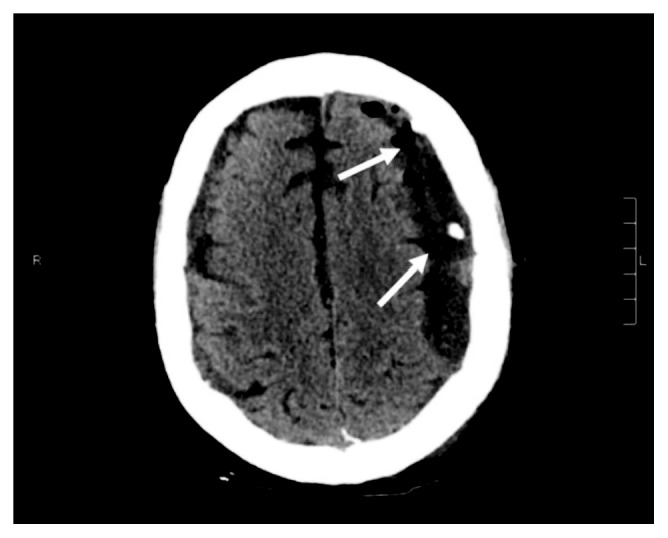
Computed tomography of the head.

A 69-year-old male with a past medical history of recently diagnosed Philadelphia-positive B-cell acute lymphoblastic leukemia (B-ALL), coronary artery disease, diabetes mellitus, and hypertension was admitted to the hospital with severe nausea and vomiting, generalized weakness, and a headache for one-day duration. The patient had been discharged from the hospital three days prior after undergoing induction treatment for a new diagnosis of B-ALL with dasatinib, vincristine, dexamethasone, and prophylactic intrathecal methotrexate. The patient completed 4 cycles of dexamethasone 40 mg daily (given as 10 mg intravenously every 6 hours) for two days each weekly cycle. The diagnosis of B-ALL was made a month prior to this hospital admission when the patient had presented with severe pancytopenia. A peripheral blood flow cytometry sent at the time of initial presentation showed positive for CD10, CD79a, CD34, and terminal deoxynucleotidyl transferase (TdT) and was negative for myeloperoxidase (MPO), CD117, and CD33. Fluorescence in situ hybridization from the bone marrow biopsy sample showed BCR-ABL translocation and gain of RUNX1T1/8q, RUNX1/21q, MYC/8q, and chromosome 5. These findings were consistent with a diagnosis of B-ALL. Due to his age and comorbidities, his treatment consisted of dasatinib 140 milligrams (mg) orally daily along with two cycles of vincristine, four cycles of dexamethasone 40 mg daily, and three cycles of prophylactic intrathecal methotrexate. Additional cycles of vincristine were not given due to cytopenias. CSF cytology samples obtained during administration of intrathecal methotrexate were negative for any involvement of ALL. The patient obtained a complete hematologic and molecular remission after his first course of treatment. He was due for his next course of intrathecal methotrexate on the day of admission but was admitted to the hospital due to the above complaints. Over the prior few weeks, he had been complaining of generalized weakness throughout his arms and his legs and gait instability associated with balance issues. He also reported intermittent left-sided headaches. On physical examination, he was afebrile with 4/5 strength in all extremities but with no cranial nerve deficits. Laboratory evaluation demonstrated the white cell count was 3.300 k/cmm, hemoglobin was 7.3 g/dL, and platelet count was 92,000 along with hypokalemia. He was initially treated supportively with intravenous fluids and antiemetics. Subsequent magnetic resonance imaging (MRI) of the brain revealed development of large bilateral extra-axial fluid collections which appeared to be subdural in location, most suggestive of subdural hygromas. There was also an associated mass effect with sulcal effacement and approximately 3 mm rightward midline shift (Figure 1). The patient denied any recent falls or trauma. His most recent lumbar puncture was performed six days prior to his current presentation. Lumbar puncture was performed under aseptic precautions following the optimized technique to avoid CSF leakage. Neurosurgery then performed a burr hole procedure with evacuation of subdural fluid with subsequent CT head showing improvement in the subdural fluid collections (Figure 2). Postoperatively, the patient's neurological status wavered. A repeat CT of the brain was obtained on postoperative day four due to new onset slurring of speech and increased lethargy but was unchanged when compared to the recent MRI of the brain. Neurosurgery placed an epidural blood patch due to suspicion of a low-pressure cerebrospinal fluid leak. He was also instructed to place his bed in the Trendelenburg position as tolerated. Repeat MRI of the brain revealed an increase in the bilateral fluid collection (Figure 3). The patient's mentation and motor function improved, and subsequent CT head showed mild improvement in the fluid collection. The patient was discharged home and presented to the oncology clinic within a week with worsening altered mental status, confusion, agitation, weakness in the lower extremities, and balance issues. Repeat CT of the head showed recurrent enlarged fluid collections (Figure 4). He had burr holes placed by neurosurgery bilaterally with subdural evacuating port systems. The fluid collections would resolve and recur over the hospital course. Repeat scans continued to show persistent mass effect, and his neurologic status continued to decline. The patient's family decided that he should be discharged with hospice, and he subsequently died a few days later.

## 3. Discussion

Acute lymphoblastic leukemia is responsible for 20% of adult leukemia cases and carries a significantly poorer prognosis than in children with overall five-year survival rates of 30–40% [4]. Among adults older than 55–60 years, the probability of survival decreases to 20% at three years [5]. With standard intensive induction chemotherapy, however, approximately 85–90% of adult patients will achieve a complete remission [6]. Patients with acute lymphoblastic leukemia (ALL) have substantial risk for developing leptomeningeal disease. In the absence of central nervous system (CNS) prophylaxis, as many as 50–75% of patients may develop CNS disease; thus, intrathecal (IT) methotrexate with or without cytarabine via lumbar puncture or Ommaya reservoir is a well-established component of induction therapy [7].

The mechanism for subdural hygroma via lumbar puncture (LP) is not well established but is presumed to involve a lumbar CSF leak with a reduction in CSF pressure, resulting in downward displacement of the brain with CSF accumulation in the inner dural layers of the cerebral convexities [8]. Bilateral subdural hygromas following prophylactic IT methotrexate is a very rare complication that has only been sporadically reported in the literature [8, 9]. A study of patients with subdural hygromas after haemopoietic stem cell transplant concluded that patients were at increased risk of developing subdural collections if they had an LP, with or without IT chemotherapy [10]. The risk of symptomatic subdural hygroma after LP is estimated to be only 1-2% [10]. In our patient, since there was no evidence for a prior structural or traumatic etiology for reduced CSF pressure, the IT administration of chemotherapy in this patient was most likely the causative mechanism of hygroma formation. In the DASISION trial, myelosuppression was commonly reported with tyrosine kinase inhibitor (TKI) therapy. Dasatinib has been known to cause serious and fatal bleeding due to platelet dysfunction, and the incidence of serious hemorrhage has been reported in a few patients, mostly associated with severe thrombocytopenia [11].

Patel et al. report an increased risk of CNS bleeding in association with tyrosine kinase inhibitor (TKI) therapy due to its effect on platelet function [12]. Though hygromas and hemorrhage are different clinical entities, subtle prohemorrhagic changes may favor the continuous meningeal leakage of CSF.

Initial management of subdural hygromas involves conservative measures, including bed rest combined with fluid supplementation, analgesic agents, and even caffeine. The epidural blood patch (EBP) is indicated if the conservative treatment is ineffective, as in our patient [13]. Other management options include an increase in platelet transfusion parameters above 50,000*–*75,000 per *μ*l, continuous epidural saline infusion, or epidural injection of dextran or fibrin glue. However, even with optimal management, the size of the subdural hygroma may increase [14, 15]. Subdural hygromas may become membrane-bound and may produce mass effect on the cerebral hemispheres requiring surgical decompression, as in our patient (Figure 1). Lumbar puncture for intrathecal therapy in these patients with cerebral mass effect may lead to fatal brain herniation.

In this report, we describe a rare but potentially fatal complication of IT chemotherapy that oncologists should be aware of. Unfortunately, our patient appeared to be a complete hematologic and molecular remission, and his death was iatrogenic, a complication from the treatment. It should be noted that IT chemotherapy is not without risk. Intrathecal chemotherapy should be deferred if any neurologic symptoms become apparent or if there are any concerning findings during the lumbar puncture, such as low opening pressures or a traumatic tap which may occur because the spinal needle is advanced beyond the collapsed subarachnoid space. Headache after lumbar puncture is a potential warning sign that has been reported by few patients prior to the diagnosis of hygroma, and if this occurs, the patient should be promptly evaluated with the consideration of imaging to rule out hygroma or hemorrhage.
